# Protective effect of the omega-3 polyunsaturated fatty acids: Eicosapentaenoic acid/Docosahexaenoic acid 1:1 ratio on cardiovascular disease risk markers in rats

**DOI:** 10.1186/1476-511X-12-140

**Published:** 2013-10-01

**Authors:** Laura Lluís, Núria Taltavull, Mònica Muñoz-Cortés, Vanesa Sánchez-Martos, Marta Romeu, Montse Giralt, Eunice Molinar-Toribio, Josep Lluís Torres, Jara Pérez-Jiménez, Manuel Pazos, Lucía Méndez, José M Gallardo, Isabel Medina, M Rosa Nogués

**Affiliations:** 1 Unit of Pharmacology, Faculty of Medicine and Health Sciences, Rovira i Virgili University, Sant Llorenç, 21, 43201 Reus, Spain; 2 Institute of Advanced Chemistry of Catalonia –CSIC (IQAC-CSIC), Barcelona, Spain; 3 Institute of Marine Research-CSIC (IIM-CSIC), Vigo, Spain

**Keywords:** Omega-3 polyunsaturated fatty acids (PUFA), Eicosapentaenoic acid (EPA), Docosahexaenoic acid (DHA), Fish oils, Oxidative stress, Antioxidant status, Cardiovascular disease risk, Insulin resistance

## Abstract

**Background:**

High consumption of fish carries a lower risk of cardiovascular disease as a consequence of dietary omega-3 long chain polyunsaturated fatty acid (n-3 PUFA; especially EPA and DHA) content. A controversy exists about the component/s responsible of these beneficial effects and, in consequence, which is the best proportion between both fatty acids. We sought to determine, in healthy Wistar rats, the proportions of EPA and DHA that would induce beneficial effects on biomarkers of oxidative stress, and cardiovascular disease risk.

**Methods:**

Female Wistar rats were fed for 13 weeks with 5 different dietary supplements of oils; 3 derived from fish (EPA/DHA ratios of 1:1, 2:1, 1:2) plus soybean and linseed as controls. The activities of major antioxidant enzymes (SOD, CAT, GPX, and GR) were determined in erythrocytes and liver, and the ORAC test was used to determine the antioxidant capacity in plasma. Also measured were: C reactive protein (CRP), endothelial dysfunction (sVCAM and sICAM), prothrombotic activity (PAI-1), lipid profile (triglycerides, cholesterol, HDLc, LDLc, Apo-A1, and Apo-B100), glycated haemoglobin and lipid peroxidation (LDL-ox and MDA values).

**Results:**

After three months of nutritional intervention, we observed statistically significant differences in the ApoB100/ApoA1 ratio, glycated haemoglobin, VCAM-1, SOD and GPx in erythrocytes, ORAC values and LDL-ox. Supplementation with fish oil derived omega-3 PUFA increased VCAM-1, LDL-ox and plasma antioxidant capacity (ORAC). Conversely, the ApoB100/ApoA1 ratio and percentage glycated haemoglobin decreased.

**Conclusions:**

Our results showed that a diet of a 1:1 ratio of EPA/DHA improved many of the oxidative stress parameters (SOD and GPx in erythrocytes), plasma antioxidant capacity (ORAC) and cardiovascular risk factors (glycated haemoglobin) relative to the other diets.

## Background

Diets with high fish content have been associated with a low cardiovascular disease (CVD) risk. Of particular note are the n-3 polyunsaturated fatty acids (n-3 PUFA): eicosapentaenoic acid (EPA, 20:5 n-3) and docosahexaenoic acid (DHA, 22:6 n-3).

Fish are the major food sources of DHA and EPA and are carried in the circulation as triglycerides, especially phospholipids [1]. There are several experimental studies that show that the n-3 PUFA perform several functions in relation to the structure and function of the membrane, tissue metabolism, and gene regulation [2]. These fatty acids play important roles in reducing hypertriglyceridaemia [3,4], low density lipoprotein cholesterol (LDLc), very low density lipoprotein cholesterol (VLDLc), and increasing high density lipoprotein cholesterol (HDLc) concentrations [5] as well as various components of these molecules e.g. ApoA1 and ApoB100 of HDL and LDL/VLDL respectively. EPA and DHA improve hypertension [6], insulin sensitivity and glycaemia [7]. Oxidative stress and vascular endothelial dysfunction also play a critical role in the pathogenesis of CVD. Increased oxidative stress underlies the pathophysiology of hypertension and atherosclerosis by directly affecting the cells of the vascular wall [8]. Higher levels of soluble intercellular adhesion molecule-1 (sICAM-1) and soluble vascular cell adhesion molecule-1 (sVCAM-1) have been associated with an increased risk of ischaemic disease and peripheral artery disease mediated, in part, by C reactive protein (CRP) [9]. Type 1 plasminogen activator inhibitor (PAI-1) is also related to metabolic syndrome, obesity, and CVD [5].

The amount of n-3 PUFA necessary to provide health benefits is unknown [10] as are the proportions of EPA and DHA that provide the greatest benefit. The majority of the clinical studies carried-out to date use fish-oil derived dietary supplements, but with a higher EPA/DHA ratio than that commonly found in the fish themselves [11,12], and it would not trigger the effects *in vivo* compared to the ratio contained in fish. EPA and DHA derived from fish oils have demonstrable cardiovascular disease benefits in observational studies and experimental trials which, mainly, have investigated their effects in combination. As such, little is known of the potentially different effects of EPA and DHA, especially regarding which has the better protective effect on CVD [2].

Hence, our present study seeks to determine the proportion of EPA/DHA that is best able to achieve a protective effect of the n-3 PUFA on CVD risk factors. Three dietary interventions with the optimal relation n-3/n-6 and different EPA/DHA ratios (1:1, 2:1, 1:2) were evaluated in a healthy animal model. Soybean and linseed oils were used as control diets. Soybean is a rich source of linoleic acid (LA, 18:2 n-6), while linseed oil has an elevated content of alpha-linolenic acid (ALA, 18:3 n-3) [13]. Parameters of oxidative stress, inflammation, endothelial dysfunction, prothrombotic state, protein glycation, lipid peroxidation, and lipid profile were determined as risk factors or biomarkers indicative of CVD risk.

## Results

### Anthropomorphic measures

None of the nutritional interventions, irrespective of the proportions of EPA/DHA (1:1, 2:1, 1:2) or soybean or linseed oil supplements, significantly increased the weight of the animals (260.3 ± 11; 259.3 ± 12; 255.5 ± 10; 259.1 ± 8; 266.3 ± 9, respectively). Abdominal fat tissue, as a percentage of overall body weight, was not significantly different in test diets compared to that of the animals on the control diets (3.89 ± 1.2; 3.62 ± 0.8; 4.12 ± 0.7; 4.3 ± 0.7; 4.2 ± 0.9, respectively).

### Antioxidant status and oxidative stress

The biomarkers of antioxidant status and oxidative stress are summarised in Table 1.

**Table 1:** Antioxidant and oxidative stress biomarkers in blood and liver of Wistar rats fed the oil supplements

**Biomarkers**	**EPA/DHA 1:1**	**EPA/DHA 2:1**		**EPA/DHA 1:2**		**Soybean oil**		**Linseed oil**	
	**Mean ± SD**	**Mean ± SD**	***p***	**Mean ± SD**	***P***	**Mean ± SD**	***p***	**Mean ± SD**	***p***
***Erythrocytes***									
SOD (U/g Hb)	2129.2 **±** 586.5	1880.9 **±** 341.2		1226.1 **±** 517.4	^a^0.023	1443.8 **±** 426.2		1230.6 **±** 283.9	^a^0.017
CAT (mmol/min/g Hb)	57.4 **±** 11.1	76.7 **±** 28.2		61.4 **±** 13.2		41.5 **±** 34.6		37.2 **±** 20.9	
GR (U/g Hb)	0.40 **±** 0.08	0.734 **±** 0.21		0.63 **±** 0.35		0.93 **±** 0.18		0.75 **±** 0.20	
GPx (U/g Hb)	107.1 **±** 15.0	116.7 **±** 30.7	^a^0.007	67.7 **±** 34.5	^a^0.026	101.5 **±** 38.2		71.1 **±** 9.6	^a^0.002
					^b^0.007				b0.001
***Plasma***									
ORAC (μmol TE/ml plasma)	48.15 **±** 16.96	23.56 **±** 5.97	^a^0.023	26.39 **±** 9.01	^a^0.016	30.32 **±** 9.64	^a^0.049	24.71 **±** 9.70	^a^0.033
LDL-ox (ng/mL)	161.53 **±** 30.9	212.25 **±** 57.4		239.43 **±** 70.02		109.03 **±** 27.34	^b^0.007	94.52 **±** 14.08	^b^0.001
							^c^0.001		^c^0.001
***Liver***									
MDA (μg MDA/ g tissue)	10.29 **±** 2.82	7.57 **±** 2.55		8.81 **±** 3.28		8.12 **±** 2.53		6.53 **±** 1.24	

The data are expressed as mean ± SD (standard deviation). ^a^: difference with respect to EPA/DHA 1:1 supplementation; ^b^: differences with respect to EPA/DHA 2:1 supplementation; ^c^: differences with respect to EPA/DHA 1:2 supplementation.

The concentrations of antioxidant enzymes in erythrocytes indicated an activation of these enzymes in fish-oil diets.

SOD values were higher in 1:1 EPA/DHA diet followed by 2:1 EPA/DHA, compared to the other 3 diets. GPx values were also higher in 1:1 and 2:1 EPA/DHA. There was a trend, albeit not statistically significant, towards higher CAT and lower GR values in fish-oil diets compared to the control diets.

These results indicated that fish-oil diets, especially 1:1 and 2:1 EPA/DHA, had improved values of antioxidant enzymes than did soybean and linseed oils.

The two control diets (soybean and linseed) had no significant differences between them with respect to the values of erythrocyte antioxidant enzymes.

Finally, the plasma antioxidant capacity (ORAC) was significantly higher in the 1:1 diet than in the other diets. This result is in agreement with the high SOD and GPx values found in this supplemented group.

### Lipid peroxidation

The mean LDL-ox values indicated higher oxidation in fish-oil diets than controls. LDL-ox of diets with 2:1 and 1:2 EPA/DHA ratios were significantly higher, compared to soybean and linseed diets. The EPA/DHA (1:1) group did not show significant differences with respect to control groups (Table 1).

Mean values of MDA in the liver were not significantly different between groups.

### Lipid profile

TG, CHOL, LDLc, HDLc, LDLc/HDLc, Apo A1 and Apo B100 were not statistically significantly different between supplemented groups (Table 2).

**Table 2:** Cardiovascular disease (CVD) risk factors in circulation in Wistar rats fed the different oil supplements

	**EPA/DHA 1:1**	**EPA/DHA 2:1**	**EPA/DHA 1:2**		**Soybean oil**		**Linseed oil**	
**Biomarkers**	**Mean ± SD**	**Mean ± SD**	**Mean ± SD**	***p***	**Mean ± SD**	***P***	**Mean ± SD**	***p***
***CV RISK FACTORS***								
***Lipid profile***								
TG (mg/dL)	86.3 **±** 65.1	124.5 **±** 102.2	83.1 **±** 10.4		108.5 **±** 51.9		73.5 **±** 25.2	
CHOL (mg/dL)	93.3 **±** 26.5	98.0 **±** 14.8	112.3 **±** 20.8		125.8 **±** 16.5		118.5 **±** 13.5	
LDLc (mg/dL)	7.64 **±** 3.3	7.4 **±** 0.8	8.30 **±** 1.3		10.39 **±** 2.2		7.4 **±** 1.6	
HDLc (mg/dL)	37.4 **±** 11.1	50.4 **±** 25.9	47.3 **±** 8.1		51.3 **±** 7.8		46.8 **±** 5.5	
LDLc/HDLc	0.19 **±** 0.5	0.20 **±** 0.01	0.17 **±** 0.01		0.20 **±** 0.2		0.19 **±** 0.1	
ApoA1 (mg/mL)	43.0 **±** 11.4	51.7 **±** 11.9	47.9 **±** 13.8		51.4 **±** 13.3		30.6 **±** 2.1	
ApoB100 (mg/mL)	116.5 **±** 16.8	137.0 **±** 6.4	139.4 **±** 23.8		126.8 **±** 13.9		152.7 **±** 35.7	
ApoB100/ApoA1	2.79 **±** 0.51	2.75 **±** 0.54	3.10 **±** 1.01		2.67 **±** 1.11		5.02 **±** 1.29	^a^0.029
								^b^0.019
								^d^0.029
***Glycaemia***								
Glucose increase (mmol/L)	-0.75 **±** 0.42	-0.15 **±** 0.34	0.27 **±** 0.49	^a^0.001	0.53 **±** 0.25	^a^ < 0.001	- 0.20 **±** 0.31	^d^0.032
Glycated Haemoglobin (%)	4.14 **±** 0.24	4.43 **±** 0.72	4.17 **±** 0.30		6.39 **±** 0.99	^a^0.003	6.34 **±** 1.84	^a^0.025
						^b^0.007		^b^0.05
						^c^0.004		^c^0.032
Insulin increase (ng/mL)	1.40 **±** 0.8	0.15 **±** 1.90	0.42 **±** 0.4		0.9 **±** 1.5		0.41 **±** 0.5	
Homa index	7.0 **±** 3.0	4.1 **±** 1.8	3.8 **±** 1.5		6.9 **±** 3.8		2.9 **±** 1.1	^a^0.004
								^d^0.032

The data are expressed as mean ± SD (standard deviation). ^a^: differences with respect to EPA/DHA 1:1 supplementation; ^b^: differences with respect to EPA/DHA 2:1 supplementation; ^c^: differences with respect to EPA/DHA 1:2 supplementation; ^d^: differences with respect to soybean oil supplementation.

TG, LDLc and HDLc values were within the reference ranges observed in other studies [14,15], whereas CHOL concentrations were increased in all the groups compared to that observed by Levy et al. [14].

The linseed-oil diet group had significantly higher values of the ApoB100/ApoA1 ratio, compared to the 1:1, 2:1 and the soybean-oil diets.

### Glycaemia control and insulin resistance

The post-intervention glucose concentrations were within the laboratory reference range in all the groups at <14 mM [16,17], but the glucose decreases obtained at the end of the experiment were greater in 1:1 and 2:1 EPA/DHA with respect to the other diets (Table 2).

All EPA/DHA diets showed significantly lower values of glycated haemoglobin, relative to the linseed and soybean oil diets. Glycated haemoglobin concentrations were not significantly different among the 3 EPA/DHA diets (Table 2).

The initial concentrations of insulin were within reference range values (3.5 – 4.4 ng/mL) described by other authors [17,18] in all groups (results not shown). The insulin increases observed at the end of the experiment were not significantly different relative to the baseline values, and without differences between groups (Table 2).

Following nutritional intervention, the Wistar rats had HOMA values within laboratory reference ranges (<14) described by other authors [19]. The linseed group had significantly lower values compared to the EPA/DHA 1:1 and soybean supplemented groups (Table 2).

### Cardiovascular disease risk biomarkers

Linseed diet decreased the sVCAM significantly with respect to the fish-oil diets while the 2:1 diet had significantly lower values than 1:2 diet (Table 3). The soybean group had lower values of PAI-1 than the 1:2 EPA/DHA group. No statistically significant differences were observed between the two control diets with respect to these biomarkers (Table 3).

**Table 3:** Cardiovascular disease (CVD) risk biomarkers in circulation in Wistar rats fed the different oil supplements

	**EPA/DHA 1:1**	**EPA/DHA 2:1**	**EPA/DHA 1:2**		**Soybean oil**		**Linseed oil**	
**Biomarkers**	**Mean ± SD**	**Mean ± SD**	**Mean ± SD**	***p***	**Mean ± SD**	***P***	**Mean ± SD**	***p***
***CVD RISK MARKERS***								
***Inflammation***								
CRP (μg/mL)	147.0 **±** 20.5	147.1 **±** 22.4	140.1 **±** 52.7		172.9 **±** 38.2		142.9 **±** 52.0	
***Thrombotic activity***								
PAI-1 (μg/mL)	7.48 **±** 1.3	8.75 **±** 1.8	9.18 **±** 1.3		6.45 **±** 0.8	^c^0.030	7.76 **±** 1.1	
***Endothelial dysfunction***								
sVCAM (μg/mL)	3.41 **±** 0.7	3.01 **±** 0.4	4.40 **±** 1.3	^b^0.046	3.36 **±** 1.7		2.10 **±** 0.4	^a^0.001
								^b^0.001
								^c^0.003
sICAM (ng/mL)	0.59 **±** 0.2	0.61 **±** 0.2	0.53 **±** 0.2		0.52 **±** 0.1		0.59 **±** 0.3	

The data are expressed as mean ± SD (standard deviation). ^a^: differences with respect to EPA/DHA 1:1 supplementation; ^b^: differences with respect to EPA/DHA 2:1 supplementation; ^c^: differences with respect to EPA/DHA 1:2 supplementation.

No significant differences were observed in CRP and in sICAM concentrations.

## Discussion

Apart from the n-3 PUFA, fish oils contain aminoacids, vitamins, selenium and other minerals which contribute to the cardiovascular benefit. The majority of studies with purified EPA or DHA have demonstrated the bioactivity and effectiveness of these fatty acids; the implication being that the substantial CVD benefit of fish oil consumption is related to the n-3 PUFA content [2]. In our study, the supplemented dose of fish oils (extrapolated to animals) provides approximately double the amount of EPA and DHA of the European Union’s recommendation for the maintenance of normal blood concentration of triglycerides in adults [20].

Soybean and linseed oils were used as control diets. Soybean is a rich source of linoleic acid (LA, 18:2 n-6), while linseed oil has an elevated content of alpha-linolenic acid (ALA, 18:3 n-3) [13].

However, all diets had a similar fat and energy content and, hence, the observed differences can be attributed to the different ratios of EPA and DHA.

Moreover, the reasoning for using a weekly administration of the oils was because in a previous test; the daily feed became very stressing for animals. According to that, it was decided a weekly doses as has been already described in the article of Méndez et al. [13].

The influence of dietary interventions on the levels and composition of plasmatic FFA was also evaluated previously in a recently reported [13] in which we demonstrated that fish oil supplementation did not change the total amount of plasmatic FFA, but it altered the profile of the individual FFA. Animals fed fish oils exhibited significantly higher levels of EPA (20:5 n-3) and DHA (22:6 n-3) compared to those fed soybean oil. The supplementation with linseed oil provided similar levels of EPA compared to those observed in the FFA fraction from animals supplemented with fish oil; however, the amount of DHA in the FFA fraction was intermediate between those supplemented with fish and those with soybean oils. Animals fed linseed oil showed the highest amount of free LA (18:3 n-3), in agreement with the elevated content of LA in the linseed oil [13].

### Oxidative stress

Fatty acids with a greater degree of unsaturation in their molecular structure are more easily oxidised. As a consequence, diets rich in fish-oils are predisposed to causing increased oxidative damage in humans and animals. However, other studies have shown that diets supplemented with fish oils do not increase cellular oxidative damage, but may even exert an antioxidant effect [21,22].

Our results indicate that improvement in activity of SOD and GPX may explain the higher plasma antioxidant capacity (ORAC) in the EPA/DHA 1:1 supplemented group. In other study, n-3 PUFA diet corrected the decreased ORAC values in diabetic rats, and was probably due to the increased erythrocyte antioxidant enzymes SOD and GPX [11]. Our results corroborate this hypothesis.

Further, these results concur with those recently reported [13] in which we demonstrated that fish-oils, especially EPA:DHA 1:1, averted protein carbonylation in plasma and liver. All these findings favour diets rich in EPA and DHA with respect to antioxidant enzymes; the proportion of 1:1 having a higher relevance than others.

### Lipid peroxidation

To date, fish-oil supplements have not been unequivocally shown to prevent oxidation of LDL particles [23]. In our study, plasma oxidised LDL was higher in the groups supplemented with fish-oil n-3 PUFA. However, EPA/DHA 1:1 showed lower values than the other two diets with n-3 PUFA, and significant differences existed only among the groups supplemented with 2:1 and 1:2 diets, relative to soybean and linseed.

LDL-ox was lower in the linseed oil group i.e. the ALA is more effective, in this case, than the administration of EPA and DHA. This is contrary to that which occurs in antioxidant protection and protein oxidation, and may be due to a synergistic effect of ALA i.e. on the one hand having less unsaturated bonds than EPA and DHA and hence less susceptible to oxidative attack while, on the other hand, although the rate of conversion from ALA to EPA and DHA is low in the organism, small quantities of these PUFA can contribute to an increase in the levels of antioxidants that protect LDL.

There were no differences observed in liver MDA among the different groups, despite differences in the consumption of n-3 PUFA. As such, although the diets rich in fish oils do not exercise a clear protection to plasma LDL oxidation, liver lipoperoxidation were not affected in these groups, relative to the soybean and linseed oil groups.

Few studies have evaluated MDA levels in liver following fish-oil administration, and the results have been contradictory. While some studies concluded that the administration of fish-oil for 2 months decrease MDA in rats with partial hepatectomy [24], other studies demonstrated, following 2 months of nutritional intervention in rats with experimental non-alcoholic fatty liver disease, that fish-oil derived n-3 PUFA increased liver MDA concentrations and promoted severe fatty liver [25]. These differences can be due to the different fatty acid proportions contained in the oils compared to other molecules with antioxidant capacity such vitamins C and E that, as well, affect the redox status of the organism.

### Lipid profile

None of the supplementations increased body-weight and the amount of abdominal fatty tissue of the animals. We need to bear in mind that the intake of total fat was the same in all groups of animals. There were trends, albeit not statistically significant, towards a decrease in triglycerides, total cholesterol, LDLc and ApoB100 in the group supplemented with EPA/DHA 1:1 diet, relative to the other groups. Decreases in these factors would support a CVD protective role for EPA/DHA 1:1 diet.

The Apo B100/Apo A1 ratio was significantly reduced by 1:1, 2:1 and soybean diets, compared to supplementation with linseed oil. Soybean and fish-oil PUFA reduce the risk of cardiovascular disease, essentially by improving the lipid profile, as has been shown by other studies [3,5].

### Glycaemia control and insulin resistance

Fish-oils are reported to be especially efficient in improving glycated haemoglobin concentrations in the circulation. Our study further highlighted that the 1:1 ratio of EPA/DHA induced the most beneficial improvement in this factor.

There is a dearth of physiological data regarding the effect of n-3 PUFA on glucose homeostasis and insulin resistance in healthy rats. Glycated haemoglobin decreased on average by 33% in the groups receiving fish-oil supplements compared to those receiving soybean and linseed diets at the end of the nutritional intervention. This result concurs with those observed by our group on the protective effect of supplementation with EPA/DHA on the carbonylation of proteins [13]. Diets enriched with fish oil decreased protein carbonylation, especially the diet with 1:1 ratio of EPA/DHA. The significant decreases in HbA1c and non-increase in circulating glucose values observed in the study, indicate a beneficial effect of marine-fish-derived long chain n-3 PUFA in healthy animals. These changes would contribute towards the prevention of some diseases such as metabolic syndrome and obesity [5,7].

In our study, all the groups had values of insulin and HOMA index within the reference range [18,19,26]; the linseed diet group showing the lowest values in these parameters. As such, the n-3 PUFA did not provoke increases in plasma insulin in healthy rats.

## Conclusions

Our results demonstrate a positive protective effect of fish-oil supplementation *in vivo*. Specifically, these beneficial protective effects depend on the different proportions of EPA and DHA; the 1:1 proportion of EPA:DHA being the most beneficial since it improved antioxidant status, oxidised LDL, Apo-B100/Apo-A1 ratio, and glycated haemoglobin.

## Methods

### Animals

This study was conducted in compliance with the norms of the Ethics Committee for Animal Research at the *Centro Superior de Investigaciones Científicas*, Spain.

Female Wistar rats (n = 35; 13 weeks of age) were purchased from Janvier (Le Genest St-Isle, France) and acclimatised for 8 days prior to the initiation of the study. The animal room was maintained at a temperature of 22 ± 2ºC and 50-60% relative humidity with a 12 h light/dark photoperiod. The animals were fed a standard pellet diet (Panlab A04, Barcelona, Spain) and had free access to bottled water and food, except for a fasting period before sacrifice.

Animals were randomly assigned to five groups (7 rats each).

### Supplementation

Three groups had dietary supplements of fish oils containing different proportions of EPA/DHA (1:1, 2:1 and 1:2). The 4^th^ group was fed soybean oil and the 5^th^ with linseed oil. Oils differing in EPA:DHA ratio were obtained by mixing appropriate quantities of the commercial fish oils. Soybean oil, obtained from unrefined organic soy oil (first cold pressing), and linseed oil, obtained from unrefined organic flax oil (first cold pressing). All diets had a similar fat and energy content [13].

Feeding the selected oil involved weekly oral doses of 0.8 mL/Kg bodyweight for 13 weeks, and administered by gavage.

Because of the high predisposition of fish oils to peroxidation, we established quality control to ensure that the oils did not oxidise during the nutritional intervention [13].

At the end of the study, the animals were anaesthetised with ketamine/xylacine (80/10 mg/kg, respectively) by intra-peritoneal injection. The animals were sacrificed by cardiac puncture, and exsanguinated. Plasma, serum and erythrocytes were stored at -80ºC until required for batched analyses. Livers were removed, washed in phosphate buffered saline, weighed, and immediately frozen in liquid nitrogen and stored at -80ºC until required for processing.

### Oxidative stress

#### ***Antioxidant enzymes in erythrocytes***

Superoxide dismutase (SOD) [27], catalase (CAT) [28], glutathione peroxidase (GPx) [29] and glutathione reductase (GR) [30] activities were determined in erythrocytes using standard spectrophotometric methods. The units of measurement for SOD, GPx and GR are expressed as U/g Hb, and as mmol/min/g Hb for CAT.

#### ***Plasma antioxidant capacity***

Plasma antioxidant capacity was measured as the oxygen radical absorbance capacity (ORAC method) [31]. The assay measures the oxidative degradation of fluorescein after being mixed with free radical generators such as azo-initiator compounds. The units of measurement are μmol trolox-equivalent/mL plasma (μmol TE/mL).

### Lipid peroxidation

#### ***Liver malondialdehyde (MDA) levels***

Liver samples were homogenised in a sodium phosphate buffer (200 mM, pH 6.25) and then ultra-centrifuged (129000 g, 1 h, 4ºC).

Lipid peroxidation was calculated by measuring MDA concentrations using high performance liquid chromatography with fluorescence detection (HPLC-FL). The results are expressed as micrograms of malondialdehyde per gram of liver [32].

#### ***Oxidised LDL***

Plasma oxidised LDL (LDL-ox) was measured using ELISA kits (CUSABIO BIOTECH, China) according to the manufacturer’s instructions. The units of measurement are expressed as ng/mL.

### Cardiovascular disease risk factors

#### ***Lipid profile***

The lipid profiles consisting of total plasma triglycerides (TG), cholesterol (CHOL), HDL cholesterol (HDLc) and LDL cholesterol (LDLc) were measured by spectrophotometric methods (SPINREACT kits, Spain). ApoA1 and ApoB100 were measured using ELISA kits (CUSABIO BIOTECH, China). The units of measurement are expressed as mg/dL for TG, CHOL, HDLc, LDLc and as mg/mL for APO A1 and APO B100.

### Glycaemia control and insulin resistance

#### ***Glucose***

Animals were fasted for 24 hours and a blood sample taken from the saphenous vein. The capillary blood was spotted on glucose strips and read in the Ascensia Elite XL glucometer (Bayer Consumer Care AG, Basel, Switzerland). The results are expressed in mmol/L.

#### ***Glycated haemoglobin***

Glycated haemoglobin was measured using a spectrophotometric kit method (SPINREACT, Spain). The units of measurement are expressed as percentage of total haemoglobin.

#### ***Insulin***

Insulin was measured using an ELISA kit (Millipore Corporation, Billerica, MA, USA). The results are expressed in ng/mL.

#### ***HOMA index***

HOMA index (Homeostasis Assessment Model) is an estimate of insulin resistance and is calculated as:

Fasting insulin (μU/mL) * fasting glucose (mmol/L) / 22.5.

### Biomarkers of cardiovascular disease risk

Plasma CRP, sICAM, sVCAM and PAI-1 were measured by ELISA kits (CUSABIO BIOTECH, China). The results are expressed as μg/mL for CRP, sVCAM and PAI-1 and in ng/mL for sICAM.

### Statistical analysis

Results were expressed as means and standard deviations for each group of dietary supplementation. The data were analysed for differences between groups using one-way analysis of variance (ANOVA). The SPSS IBM 19 for Windows was used throughout. When significant differences were found, the means were compared using the Scheffé *post-hoc* test. Statistical significant was set at p < 0.05.

## Competing interests

The authors declare that they have no competing interests.

## Authors’ contributions

All the authors have contributed substantially to the design and execution of the study as well as drafting and revising the manuscript. All have approved the final version submitted for publication.

## References

[B1] Ackman RG Biogenic Lipids Fats and Oils. Boca Ratón 1989 Florida: CRC Press

[B2] Mozaffarian D Wu JH (n-3) fatty acids and cardiovascular health: are effects of EPA and DHA shared or complementary? J Nutrdoi:10.3945/jn.111.149633 10.3945/jn.111.149633 PMC3278271 22279134

[B3] Aguilera AA Diaz GH Barcelata ML Guerrero OA Ros RM Effects of fish oil on hypertension, plasma lipids, and tumor necrosis factor-alpha in rats with sucrose induced metabolic syndrome J Nutr Biochem 2004 15 350 357 10.1016/j.jnutbio.2003.12.008 15157941

[B4] Dyerberg J Platelet - vessel wall interaction: Influence of diet Philos Trans R Soc Lond B Biol Sci 1981 294 373 381 10.1098/rstb.1981.0113 6117898

[B5] Poudyal H Panchal SK Diwan V Brown L Omega-3 fatty acids and metabolic syndrome: Effects and emerging mechanisms of action Prog Lipid Res 2011 50 372 387 10.1016/j.plipres.2011.06.003 21762726

[B6] Armitage JA Pearce AD Sinclair AJ Vingrys AJ Weisinger RS Weisinger HS Increased blood pressure later in life may be associated with perinatal n-3 fatty acid deficiency Lipids 2003 38 459 464 10.1007/s11745-003-1084-y 12848294

[B7] Sener A Zhang Y Bulur N Louchami K Malaisse WJ Carpentier YA The metabolic syndrome of omega3-depleted rats. II. Body weight, adipose tissue mass and glycemic homeostasis Int J Mol Med 2009 24 125 129 19513544

[B8] Nakazono K Watanabe N Matsuno K Sasaki J Sato T Inoue M Does superoxide underlie the pathogenesis of hypertension? Proc Natl Acad Sci USA 1991 88 10045 10048 10.1073/pnas.88.22.10045 1658794 PMC52864

[B9] Urso C Hopps E Caimi G Adhesion molecules and diabetes mellitus Clin Ter 2010 161 e17 e24 20544149

[B10] Dubnov G Berry EM Omega-6/omega-3 fatty acid ratio: the Israeli paradox World Rev Nutr Diet 2003 92 81 91 14579685 10.1159/000073794

[B11] Ginty AT Conklin SM Preliminary evidence that acute long-chain omega-3 supplementation reduces cardiovascular reactivity to mental stress: a randomized and placebo controlled trial Biol Psychol 2012 89 269 272 10.1016/j.biopsycho.2011.09.012 21967854

[B12] Schuchardt JP Neubronner J Kressel G Merkel M von Schacky C Hahn A Moderate doses of EPA and DHA from re-esterified triacylglycerols but not from ethyl esters lower fasting serum triacylglycerols in statin-treated dyslipidemic subjects: results from a six month randomized controlled trial Prostaglandins Leukot Essent Fatty Acids 2011 85 381 386 10.1016/j.plefa.2011.07.006 21862301

[B13] Méndez L Pazos M Gallardo JM Torres JL Pérez-Jimenez J Nogués R Romeu M Medina I Reduced protein oxidation in Wistar rats supplemented with marine ω-3 PUFAs Free Radic Biol Med 2013 55 8 20 23159545 10.1016/j.freeradbiomed.2012.11.004

[B14] Levy E Brunet S Alvarez F Seidman E Bouchard G Escobar E Martin S Abnormal hepatobiliary and circulating lipid metabolism in the Long-Evans Cinnamon rat model of Wilson’s disease Life Sci 2007 80 1472 1483 10.1016/j.lfs.2007.01.017 17303181

[B15] Rusinol AE Lysak PS Sigurdson GT Vance JE Monomethylethanolamine reduces plasma triacylglycerols and apolipoprotein B and increases apolipoprotein A-I rats without induction of fatty liver J Lipid Res 1996 37 2296 2304 8978481

[B16] Yessoufou A Soulaimann N Merzouk SA Moutairou K Ahissou H Prost J Simonin AM Merzouk H Hichami A Khan NA N-3 fatty acids modulate antioxidant status in diabetic rats and their macrosomic offspring Int J Obes (Lond) 2006 30 739 750 10.1038/sj.ijo.0803211 16418759

[B17] El-Seweidy MM Sadik NA Shaker OG Role of sulfurous mineral water and sodium hydrosulfide as potent inhibitors of fibrosis in the heart of diabetic rats Arch Biochem Biophys 2011 506 48 57 10.1016/j.abb.2010.10.014 20965145

[B18] Hassanali Z Ametaj BN Field CJ Proctor SD Vine DF Dietary supplementation of n-3 PUFA reduces weight gain and improves postprandial lipaemia and the associated inflammatory response in the obese JCR: LA-cp rat Diabetes Obes Metab 2010 12 139 147 10.1111/j.1463-1326.2009.01130.x 19917068

[B19] Shah KB Duda MK O’Shea KM Sparagna GC Chess DJ Khairallah RJ Robillard-Frayne I Xu W Murphy RC Des Rosiers C Stanley WC The cardioprotective effects of fish oil during pressure overload are blocked by high fat intake: Role of cardiac phospholipid remodeling Hypertension 2009 54 605 611 10.1161/HYPERTENSIONAHA.109.135806 19597033 PMC3103889

[B20] EFSA Scientific Opinion on the substantiation of health claims related to eicosapentaenoic acid (EPA), docosahexaenoic acid (DHA), docosapentaenoic acid (DPA) and maintenance of normal cardiac function, maintenance of normal blood glucose concentrations, maintenance of normal blood pressure, maintenance of normal blood HDL-cholesterol concentrations, maintenance of normal (fasting) blood concentrations of triglycerides EFSA J 2010 8 1796

[B21] Romieu I Garcia-Esteban R Sunyer J Rios C Alcaraz-Zubeldia M Velasco SR Holguin F The effect of supplementation with omega-3 polyunsaturated fatty acids on markers of oxidative stress in elderly exposed to PM (2.5) Environ Health Perspect 2008 116 1237 1242 10.1289/ehp.10578 18795169 PMC2535628

[B22] Grim JM Hyndman KA Kriska T Girotti AW Crockett EL Relationship between oxidizable fatty acid content and level of antioxidant glutathione peroxidases in marine fish J Exp Biol 2011 214 3751 3759 10.1242/jeb.058214 22031739 PMC3202513

[B23] Lapointe A Couillard C Lemieux S Effects of dietary factors on oxidation of low density lipoprotein particles J Nutr Biochem 2006 17 645 658 10.1016/j.jnutbio.2006.01.001 16517144

[B24] Kirimlioglu V Kirimlioglu H Yilmaz S Ozgor D Coban S Karadag N Yologlu S Effect of fish oil, olive oil, and vitamin E on liver pathology, cell proliferation, and antioxidant defense system in rats subjected to partial hepatectomy Transplant Proc 2006 38 564 567 10.1016/j.transproceed.2006.02.005 16549176

[B25] Hussein O Grosovski M Lasri E Svalb S Ravid U Assy N Monounsaturated fat decreases hepatic lipid content in non-alcoholic fatty liver disease in rats World J Gastroenterol 2007 13 361 368 17230603 10.3748/wjg.v13.i3.361 PMC4065889

[B26] Ferreira L Teixeira-de-Lemos E Pinto F Parada B Mega C Vala H Pinto R Garrido P Sereno J Fernandes R Santos P Velada I Melo A Nunes S Teixeira F Reis F Effects of sitagliptin treatment on dysmetabolism, inflammation, and oxidative stress in an animal model of type 2 diabetes (ZDF rat) Mediators Inflamm 10.1155/2010/592760 10.1155/2010/592760 PMC2905949 20652060

[B27] Misra HP Fridovich I The role of superoxide anion in the autoxidation of epinephrine and a simple assay for superoxide dismutase J Biol Chem 1972 247 10 3170 3175 4623845

[B28] Cohen G Dembiec D Marcus J Measurement of catalase activity in tissue extracts Anal Biochem 1970 34 30 38 10.1016/0003-2697(70)90083-7 5440916

[B29] Wheeler CR Salzman JA Elsayed NM Omaye ST Korte DW Jr Automated assays for superoxide dismutase, catalase, glutathione peroxidase, and glutathione reductase activity Anal Biochem 1990 184 193 199 10.1016/0003-2697(90)90668-Y 2327564

[B30] Goldberg DM Spooner RJ Bergmeyer HU Glutathione reductase Methods of enzymatic analysis 1983 3 Weinheim, Germany: Verlag Chemie 258 265

[B31] Cao G Booth SL Sadowski JA Prior RL Increases in human plasma antioxidant capacity after consumption of controlled diets high in fruit and vegetables Am J Clin Nutr 1998 68 1081 1087 9808226 10.1093/ajcn/68.5.1081

[B32] Mateos R Lecumberri E Ramos S Goya L Bravo L Determination of malondialdehyde (MDA) by high-performance liquid chromatography in serum and liver as a biomarker for oxidative stress. Application to a rat model for hypercholesterolemia and evaluation of the effect of diets rich in phenolic antioxidants from fruits J Chromatogr B Analyt Technol Biomed Life Sci 2005 827 76 82 10.1016/j.jchromb.2005.06.035 16009604

